# CCR9/CCL25 expression in non-small cell lung cancer correlates with aggressive disease and mediates key steps of metastasis

**DOI:** 10.18632/oncotarget.2526

**Published:** 2014-09-25

**Authors:** Pranav Gupta, Praveen K. Sharma, Hina Mir, Rajesh Singh, Nalinaksha Singh, Goetz H. Kloecker, James W. Lillard, Shailesh Singh

**Affiliations:** ^1^ Morehouse School of Medicine, Atlanta, GA, USA; ^2^ School of Natural Sciences, Center of Life Sciences, Central University of Jharkhand, Ranchi, India; ^3^ James Graham Brown Cancer Center, University of Louisville, School of Medicine, Louisville, KY, USA

**Keywords:** CCR9, CCL25, Chemokine receptor, Non-small cell lung cancer

## Abstract

Poor clinical outcome of lung cancer (LuCa) is primarily due to lack of knowledge about specific molecules involved in its progression and metastasis. In this study, we for the first time show the clinical and biological significance of CC chemokine receptor-9 (CCR9) in non-small cell lung cancer (NSCLC). Expression of CCR9 and CCL25, the only natural ligand of CCR9, was significantly higher (p < 0.0001) in NSCLC tissues and serum respectively, compared to their respective controls. Interestingly, expression of both CCR9 and CCL25 was significantly higher in adenocarcinomas (ACs) compared to squamous cell carcinomas (SCCs) (p = 0.04, and p < 0.0001). Similar to tissues, AC and SCC cell lines were positive for CCR9 expression. Despite of marginal difference in CCR9 expression, AC cells showed higher migratory and invasive potential in response to CCL25, compared to SCC cells. This differential biological response of AC cells was primarily due to differential expression of matrix metalloproteinases and tissue inhibitor of metalloproteinases under the influence of CCL25. Our results suggest CCR9 as a potential target for developing new treatment modality for NSCLC. Additionally, differential serum CCL25 level in ACs and SCCs, two NSCLC subtypes, suggest its potential as a non-invasive diagnostic/prognostic biomarker.

## INTRODUCTION

Lung cancer (LuCa) is a leading cause of death for both men and women worldwide, with an estimated 159,260 deaths (86,930 men and 72,330 women) in the United States in 2014 (American Cancer Society). Despite advance treatment and surgical options offered in the clinics, high incidence, limited screening, and rapid progression of the disease lead to a poor prognosis and quality of life [1-3]. LuCa is classified into two major types, non-small lung cancer (NSCLC) and small cell lung cancer (SCLC). NSCLC, which accounts for 85% of lung cancer cases, have two most common subtypes i.e. squamous cell carcinoma (SCC) and adenocarcinoma (AC). The latter form of the disease accounts for about 40% of all NSCLC cases and has a poorer prognosis relative to SCC [1,4]. Due to metastatic relapse, patients diagnosed with NSCLC have an average of only five years of survival [4,5]. Nevertheless, the molecular mechanisms involved in the metastatic process and factors involved in disseminating primary tumor and directing disseminated cells to specific organs are not fully elucidated. Notably, this process shares similarities with immune cell trafficking, which is primarily mediated by chemokine-chemokine receptors.

Chemokines and their seven trans-membrane G-protein coupled receptors are known for their roles in inflammation, leukocyte trafficking, and immune differentiation. Many cancer cell types specifically express different CC and CXC chemokine-chemokine receptors, including CCR6, CCR9, CXCR4, and CXCR5 [6]. CCR9 and its natural ligand, CCL25, which is a thymus-expressed chemokine, are primarily involved in immune homeostasis [5,7-10]. However, different studies have shown the involvement of CCR9-CCL25 axis in colorectal, prostate, ovarian and breast cancers [7-11]. In patients with breast or ovarian carcinomas, higher CCR9 expression in cancer tissues correlate with disease severity, and CCR9 expressing cancer cells migrate and invade under a chemotactic gradient of CCL25 via up-regulation of matrix metalloproteinases (MMPs) [10-12]. Further, we and others have shown that CCR9-CCL25 interaction supports cancer cell survival by inhibiting chemotherapy-induced apoptosis in a PI3K-/Akt-dependent and focal adhesion kinase (FAK)-independent manner [4,12-17]. Although few studies have addressed the significance of chemokine/chemokine receptor expression in NSCLC, it has been suggested that, for NSCLC, higher expressions of CXCR1, CXCR2, and CXCR4 with their ligands CXCL5, CXCL8, and CXCL12 are associated with tumor angiogenesis, metastasis, and poor survival [2-4,11,13-17].

In this report, for the first time we have shown higher expression of CCR9 and/or CCL25 in clinical samples and cell lines from NSCLC. In addition to the clinical relevance of CCR9/CCL25 expression, we also show the biological significance of this axis in cultured LuCa cells. Our findings suggest that CCR9-CCL25 interaction aids in LuCa progression by facilitating the migration, invasion, and metastasis of LuCa cells, and that blocking this axis would inhibit LuCa metastasis and progression. In addition to this, significantly higher CCR9/CCL25 in AC patients than SCC suggests its association with aggressive disease and could be used as potential prognostic biomarker.

## RESULTS

### CCR9 is expressed in LuCa tissues and correlates with tumor stage

**Figure 1:**
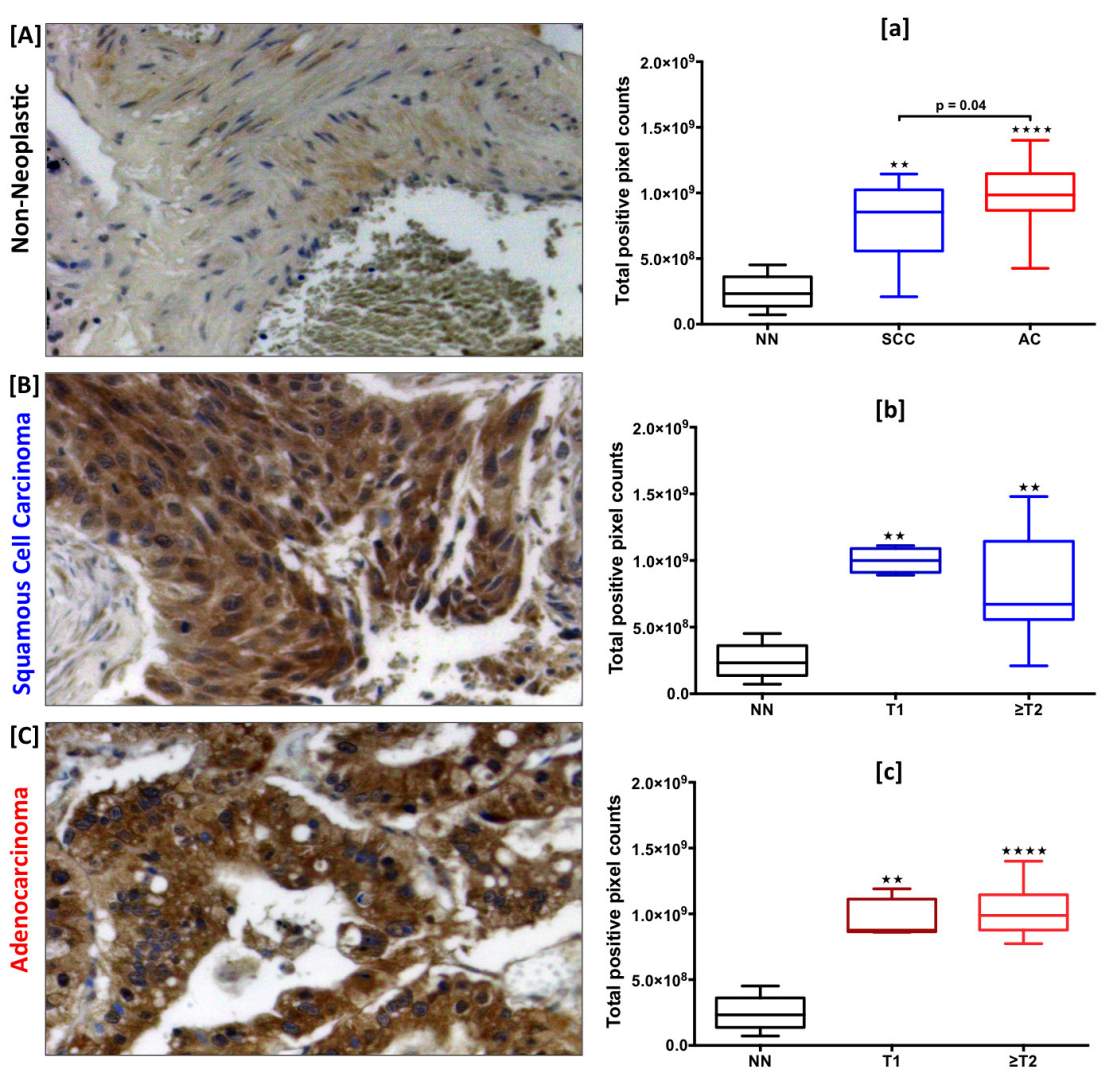
Expression of CCR9 in LuCa tissues Representative figures of [A] non-neoplastic (n=8), [B] SCC (n=12) and [C] AC (n=25) lung tissues stained with anti-CCR9 antibodies. Brown (DAB) color shows CCR9 staining. The images were captured with TissueFAXS tissue analysis system using a 20X objective. Immuno-intensities of CCR9 in each section were quantified with HistoFAXS tissue analysis system. [a] CCR9 expression in non-neoplastic (NN, n=8), SCC (n=12), and AC (n=25) tissues. Asterisks show significant differences (** p < 0.01; **** p < 0.0001) between lung cancer and control groups. [b and c] Expression of CCR9 with respect to tumor stages in SCC and AC cases, respectively. Asterisks show significant differences (** p < 0.01; **** p < 0.0001) between NN vs. T1 or ≥T2 in SCC and AC groups. All the statistical analyses were done by Mann Whitney U test. Box plots in each figure show the minimum and maximum values and the lines in the box plots indicate the median CCR9 expression of each group.

Expression of CCR9 was higher in SCC and AC tissues compared to non-neoplastic tissues (Fig. 1A, B, and C). CCR9 expression, presented in terms of total positive pixel counts, was highest in AC with a median value of 9.8×10^8^, followed by SCC with a median value of 8.7×10^8^, relative to non-neoplastic tissues with a median value of 2.3×10^8^ (p < 0.0001 and p < 0.01 respectively) (Fig. 1a). Interestingly, ACs showed significantly higher CCR9 expression compared to SCCs (p = 0.04). Further, CCR9 expression was correlated with tumor stages (T) for SCC and AC tissues. In SCCs, expression of CCR9 in stage ≥T2 (median value 6.7×10^8^) was lower than in stage T1 (median value 9.9×10^8^). However, the maximum value was higher in ≥T2 (1.5×10^9^) than in T1 (1.1×10^9^). CCR9 expression in ≥T2 ACs (median value 9.9×10^8^) was higher than in T1 (median value 8.8×10^8^) (Fig. 1b and c).

### Serum CCL25 level is elevated in LuCa patients

**Figure 2:**
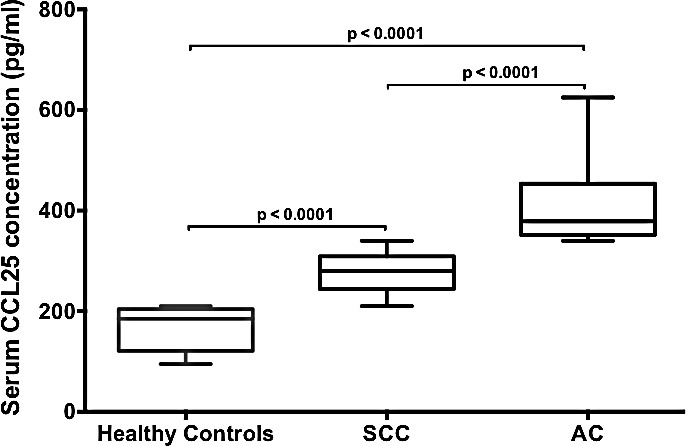
Serum CCL25 levels in LuCa patients Serum CCL25 levels in healthy donors (n=9), SCC (n=17) and AC (n=14) patients were analyzed by ELISA. As used, the ELISA could detect >5 pg/mL of CCL25. Box plots for each group show the minimum and maximum values. The lines in the box plots indicate the median serum CCL25 concentrations of each group. The difference in CCL25 concentrations between healthy controls and SCC or AC and SCC and AC were analyzed using Mann Whitney U test and were highly significant (p < 0.0001).

Serum CCL25 levels in SCC and AC patients and in healthy controls were quantified by ELISA. Level of CCL25 was highest in AC patients, followed by SCC patients, relative to healthy controls (Fig. 2). The median values of serum CCL25 in SCC and AC patients were 280 and 378 pg/ml, respectively, whereas CCL25 expression in sera from healthy controls was lower, with a median value of 185 pg/ml. All these comparisons (healthy controls vs. SCC or AC, and SCC vs. AC) were statistically significant (p < 0.0001). Higher serum CCL25 in AC patients compared to SCC suggests the significance of CCL25 as a potential prognostic indicator.

### CCR9 is expressed in LuCa cell lines and mediates cell migration and invasion

**Figure 3:**
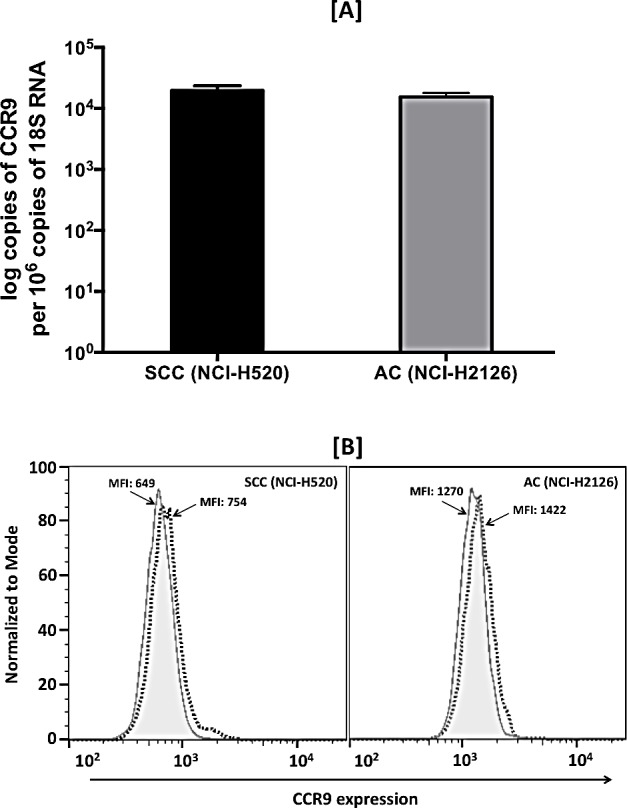
Expression of CCR9 in LuCa cell lines [A] Total RNA was isolated from SCC (NCI-H520) and AC (NCI-H2126) cells. Quantitative RT-PCR analysis of CCR9 mRNA expression was performed in triplicate. The copies of transcripts are expressed relative to copies of 18S rRNA mean +/− S.D., n=2. The asterisk indicates statistical significance (p = 0.0432) between SCC and AC cells. [B] SCC (NCI-H520) and AC (NCI-H2126) cells were stained with PE-conjugated isotype control antibodies (solid histogram) or PE-conjugated anti-CCR9 monoclonal antibodies (dashed histogram) and quantified by flow cytometry. The mean fluorescent intensities (MFIs) of PE-positive cells are shown.

Expression of CCR9 mRNA and protein in SCC (NCI-H520) and AC (NCI-H2126) cell lines was evaluated by quantitative RT-PCR and flow cytometry, respectively. CCR9 mRNA copy number per 10^6^ copies of 18S RNA were 2.1×10^4^ and 1.94×10^4^ in SCC and AC cells, respectively (Fig. 3A). Consistent with results for mRNA expression, both cell lines stained positive for CCR9. The intensity of membrane CCR9 expression, measured in terms of mean fluorescence intensity, was similar in SCC and AC cells. There were higher expressions, 1.16 and 1.12 fold, of CCR9 in SCC and AC cells, respectively, relative to their isotype intensities (Fig. 3B). No CCL25 mRNA expression could be detected in the LuCa cell lines, which suggests that CCR9 is not activated in an autocrine fashion.

**Figure 4:**
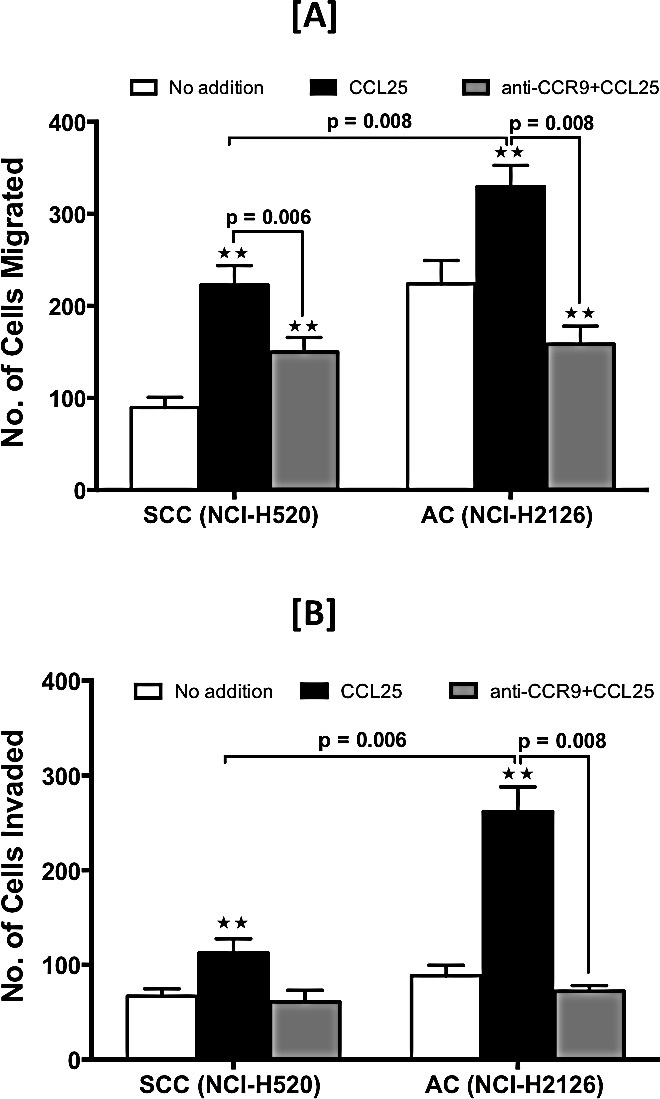
LuCa cell migration and invasion mediated by CCR9-CCL25 SCC (NCI-H520) and AC (NCI-H2126) cells were tested for their ability to migrate [A] and invade [B] toward chemotactic gradients of 0 (open bar) or 100 ng/ml (black bar) of CCL25. The cells were pre-treated with anti-human CCR9 antibody (1 μg/ml) (grey bar) during migration and invasion assays. Asterisks indicate significant differences in migration and invasion between untreated and CCL25-treated or anti-CCR9-treated cell lines (** p < 0.01). Data was analyzed using non-parametric two tailed t-test and is presented as mean +/− S.D., n=3.

The function of CCR9 in LuCa cells was demonstrated by determining the capacity of SCC and AC cells to migrate towards a chemotactic gradient of CCL25, the natural ligand for CCR9. Relative to controls, higher number of SCC and AC cells migrated, and invaded through Matrigel under the chemotactic gradient of CCL25 (Fig. 4A and 4B). The number of AC cells that migrated and invaded in response to CCL25 was higher than that of SCC cells, and in each case the process was inhibited by anti-CCR9 antibodies. These results, which were significant (p < 0.01), demonstrate that CCR9-CCL25 axis is functional in LuCa cells.

### CCL25 modulates expression of Matrix Metalloproteinases (MMPs) and Tissue Inhibitor of Metalloproteinases (TIMPs) in LuCa cell lines

**Figure 5:**
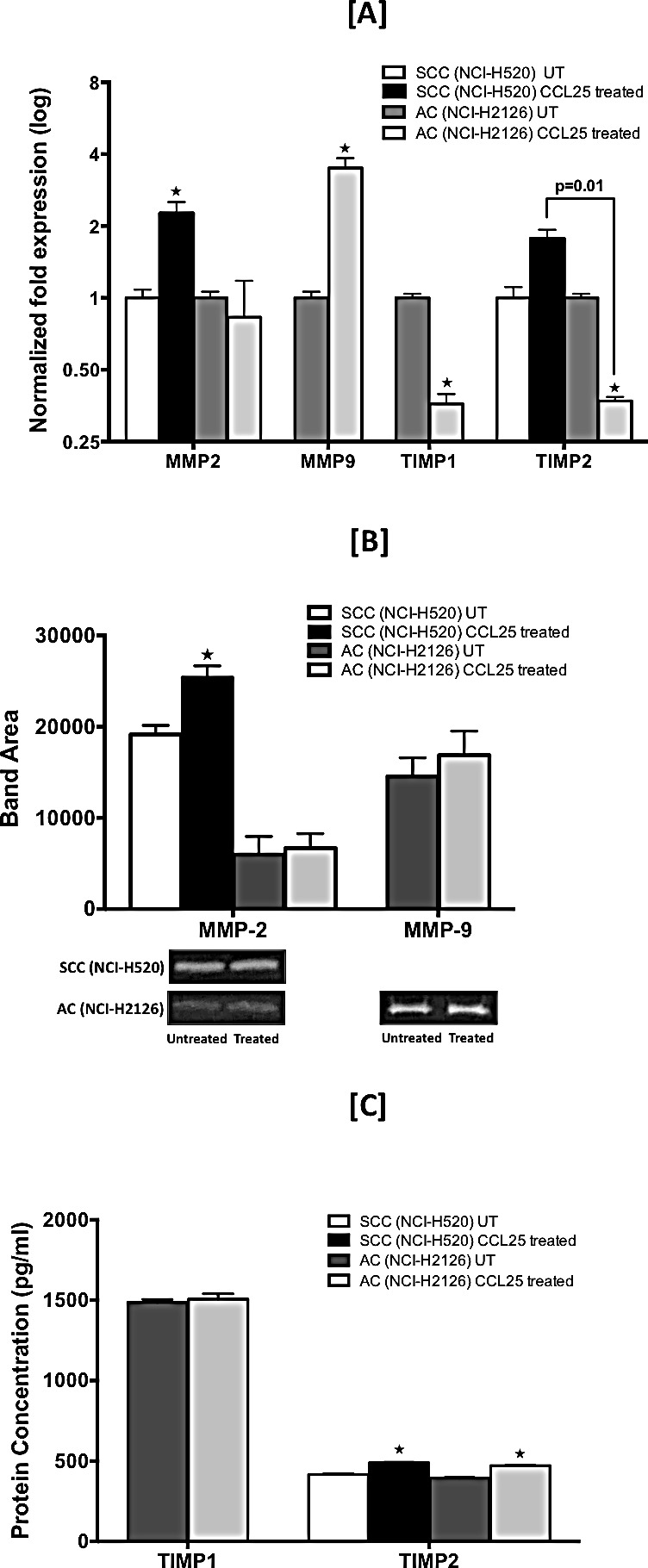
CCL25-induced expression of MMPs and TIMPs in LuCa cells Cells were tested for their capacity to express mRNA and protein for MMP-2 and -9 and TIMP-1 and -2. [A] SCC (NCI-H520) and AC (NCI-H2126) cells were treated for 30 min with 0 or 100 ng/mL of CCL25. Total RNA was isolated, and quantitative real time-PCR analysis was performed for mRNA expression of MMP-2 and -9 and TIMP-1 and -2. Transcript copies were presented relative to copies of 18S rRNA. [B] Active gelatinases (MMP-2 and -9) in culture supernatants were quantified by gelatin zymography. Cells were stimulated with CCL25 (0 or 100 ng/ml) for 24 h. Top: Graph represents densitometric analysis of zymography for control and treated samples, presented as band area, analyzed by ImageJ software. Bottom: Representative zymography. [C] TIMP-1 and TIMP-2 in culture supernatants were quantified by ELISA. Bars represent the concentration (pg/ml) of TIMP-1 and TIMP-2 in culture supernatants collected from cells treated with 0 or 100 ng/ml of CCL25 for 24 h. Asterisks show significant differences between untreated and CCL25-treated LuCa cells. Data was analyzed by non-parametric two tailed t-test and presented as mean +/− S.D., n=2. * p < 0.05.

To determine the underlying mechanism behind CCR9-CCL25-mediated migration and invasion of LuCa cells, expressions of MMPs and TIMPs in these cells were analyzed following treatment with CCL25. The level of MMP-2 mRNA was increased following CCL25 treatment by 2.25 fold and 1.1 fold in SCC and AC cells, respectively (Fig. 5A). Expression of MMP-9 mRNA in AC cells was increased by 3.5 fold in response to CCL25 treatment. In SCC cells, however, expression of MMP-9 was below the range of detection. There were no major changes in the expression of other MMPs in either of the LuCa cell lines following CCL25 treatment (data not shown). There was 1.7-fold increase in the expression of TIMP-2 mRNA in response to CCL25 treatment in SCC cells relative to that in untreated cells. In AC cells treated with CCL25, expressions of both TIMP-1 and TIMP-2 mRNAs were decreased to 0.36 fold relative to amounts in untreated cells.

The effect of CCL25 on active MMP-2 and MMP-9 secretion by LuCa cell lines was determined by gelatin zymography (Fig. 5B). Significantly higher MMP-2 activity, presented as band area, was observed in culture supernatants from SCC cells stimulated with CCL25 relative to untreated controls (25432 vs. 19146) (p < 0.05). Similar to mRNA expression results, no MMP-9 activity could be detected in samples from SCC cells. Both MMP-2 and MMP-9 activity was observed in culture supernatants from AC cells, and both activities were higher in CCL25-treated samples relative to untreated controls (5974 vs. 6674 for MMP-2 and 14562 vs. 16874 for MMP-9).

Levels of TIMP-1 and TIMP-2 proteins in culture supernatants after CCL25 treatment were determined by ELISA; samples from untreated cells were used as controls. Both TIMP-1 and TIMP-2 proteins were quantified in AC cells, but TIMP-1 protein could not be detected in either treated or untreated samples of SCC cells. Although, relative to controls, there was no significant change in TIMP-1 levels (1487 pg/ml vs. 1507 pg/ml) in AC cells, TIMP-2 was significantly elevated (395 pg/ml vs. 471 pg/ml) in response to CCL25 (p < 0.05). Similar results were observed for TIMP-2 levels in SCC cells (417 pg/ml vs. 489 pg/ml) (p < 0.05).

## DISCUSSION

Chemokines and their corresponding receptors are primarily known to be involved in leukocyte trafficking and host defense. However, recent evidences suggest that tumor cells acquire this skill of immune cells and exploite the chemokine and their corresponding receptors for their survival and dissemination, and use them as a navigational tool to find secondary sites for metastasis [18-21]. Among all chemokine receptors known to be involved in the progression of different cancers including LuCa, CXCR4 is expressed in majority of cancers and plays an important role in dissemination and homing of primary tumor at distant sites [6,22-25]. Other than CXCR4, we and other groups have also shown the involvement of CXCR5 and CCR9 in survival and metastasis of tumor cells in different malignancies [8,11,12,26-28]. In this study for the first time we have shown the potential role of CCR9 in LuCa, which was highly expressed in non-small cell lung carcinoma (NSCLC) tissues compared to controls. Interestingly, serum CCL25, which is the only natural ligand for CCR9, was also significantly elevated in NSCLC patients compared to controls, suggesting the clinical significance of CCR9/CCL25 in LuCa. Lung AC patients have poorer prognosis compared to SCC [29,30]; higher CCR9/CCL25 expression in AC patients compared to SCC observed in our study suggest possible association of CCR9-CCL25 axis with LuCa aggressiveness, and holds promise to be validated as prognostic indicator in LuCa. Clinical staging of cancer is based on TNM, which is used to determine the clinical outcome, determining therapeutic interventions and prognosis [31]. Patients with higher tumor stage tend to have poorer prognosis [32]. Interestingly, higher expression of CCR9 in NSCLC correlated with tumor size (T); higher tumor size is often associated with metastatic tumor. We have previously shown that CCR9 and CCL25 play significant role in prostate cancer cell survival, which is required for cancer cells to achieve their metastatic goal [8]. Hence, higher expression of CCR9 in NSCLC suggests its potential role in dissemination of primary tumor and promoting tumor cell survival during metastasis. Subsequent studies with higher number of patients in each group (SCC and AC) and subgroups (T1 and ≥T2) should provide conclusive information correlating CCR9 expression in NSCLC with disease progression and survival.

We and others have shown the clinical and biological significance of CCR9-CCL25 axis in various solid tumors, e.g., prostate, ovarian, colorectal, and breast cancers [8,11,33,34]. In this study, we have shown the biological significance of this axis using NSCLC cell lines derived from SSC (NCI-H520) and AC (NCI-H2126). Similar to tissue expression, CCR9 was expressed by NSCLC cell lines. Despite of marginal difference in CCR9 expression, AC cell lines showed higher invasive and metastatic potential compared to SCC cells, which was evident in *in vitro* assays, performed under the chemotactic gradient of CCL25. Chemokine receptors mediated signaling is very much dependent on their internalization, recycling and/or degradation. Chemokine receptors after binding of their ligands/agonists cluster into clathrin-coated domains of the plasma membrane [35]. Hence, higher biological responses in AC cells compared to SCC cells, with both having similar CCR9 expression, could be associated with post ligation modifications in CCR9, recycling and/or phosphorylation of CCR9 following CCL25 stimulation in AC cells. Furthermore, tumor cells make MMPs, which digest the basement membrane and facilitate tumor cell invasion to new tissues. Although various MMPs have been implicated in acquisition of invasive and metastatic properties by tumor cells, MMP-2 and MMP-9, which degrade type IV collagen, a major component of basement membranes, are majorly associated with metastasis [36-39]. It was interesting to note that AC cells were making both MMP-2 and -9 under the influence of CCL25, whereas SCC cells were making only MMP-2. Furthermore, expression and activity of MMP-2 in response to CCL25 was higher in AC cells compared to SCC. Hence, differential expression and activities of MMPs, presumably due to the differences in CCR9 recycling/phosphorylation, produced by these cells following CCL25 stimulation could be responsible for their differential biological activities.

TIMPs inhibit the activities of MMPs and have been considered as anti-cancer proteins, however recent studies have demonstrated a contradictory pro-tumor role of TIMPs [40-43]. Elevated plasma levels of TIMP-1 are associated with worse clinical outcomes of colon or prostate cancer patients [44]. Further, TIMP-2 over-expression stimulates proliferation of human osteosarcoma [45] and A549 lung AC cells [46,47] and protects melanoma cells from apoptosis by modulating the NF-κB pathway [43]. TIMPs affect cancer progression in both MMP-dependent and MMP-independent manner [48,49]. Hence, higher TIMP expression by LuCa cells in response to CCL25 suggests potential involvement of CCR9-CCL25 axis in LuCa progression and outcome. Interestingly, AC cells make both TIMP-1 and TIMP-2, while only TIMP-2 was detected in SCC. This further suggests that differential expression of MMPs and TIMPs in AC cells following CCL25 stimulation is involved in maintaining the aggressive phenotype and can be correlated with the poorer prognosis of AC cases.

In conclusion, elevated serum CCL25 in AC patients and differential expression of CCR9 in AC tissues suggests the clinical and prognostic significance of CCR9-CCL25 axis in LuCa. Higher biological response and selective modulation of key metastatic factors (MMPs and TIMPs) in AC cells following CCL25 treatment, further suggest that this chemokine-receptor axis play crucial role in LuCa metastasis and maintaining aggressive phenotype. Hence, blocking CCR9-CCL25 axis may improve the therapeutic outcome and overall survival of LuCa patients.

## METHODS

### Tissue specimens

Tissue microarray (TMA) slides containing malignant (n = 45) and non-neoplastic (n = 8) samples were procured from AccuMax Array (ISU Abxis Co., Ltd.). These were generated from lung biopsies of 39 cases diagnosed with NSCLC with histological subtypes of AC (n = 27), SCC (n = 12), and others (n = 6); and 8 non-neoplastic cases. To construct the TMA slides, two cores (1 mm in diameter) per patient were arrayed on a blank paraffin block, and a qualified pathologist validated the histopathology of each core twice for class and grade of the tumor.

### Immunohistochemistry and quantitation of immunohistochemical staining

TMA slides containing malignant and non-neoplastic tissues were stained for CCR9. Briefly, TMAs were de-paraffinized in xylene and rehydrated through a graded series of ethanol (100%, 95% and 70%) for 5 min in each series and washed in distilled water. Following de-paraffinization, antigen retrieval was performed by incubating TMAs with 0.01 M EDTA (pH 8.0) in a pressure cooker for 5 min. Slides were then cooled in running water and transferred to Tris-buffer (pH 7.6). The endogenous peroxidase activity was blocked by incubating the slides with 3% H_2_O_2_ in PBS for 5 min. The slides were then rinsed three times each with de-ionized water, followed by Tris-buffer (pH 7.6). Fc blocking was accomplished by incubating slides with Fc block (Innovex Biosciences, CA, USA) for 30 min at room temperature (RT) in a humidity chamber. To reduce non-specific binding, the sections were washed with Tris-buffer and incubated with 3% normal goat serum for 1 h at RT. The slides were then washed with Tris-buffer, and sections were incubated with 5 μg/ml of HRP-conjugated mouse anti-CCR9 antibody (R&D Systems, MN) for 90 min at RT in a humidity chamber. The negative control slide was incubated with 5 μg/ml mouse isotype control antibody (R&D Systems). Following incubation, sections were washed with Tris buffer and developed with 3,3′-diaminobenzidine (DAB) substrate kits (Vector Laboratories, CA) for 25 min at RT. Counterstaining was accomplished with hematoxylin (Sigma, MO). Subsequently, sections were washed with water, dehydrated in 70%, 95%, and absolute alcohol for 5 min each; passed through xylene three times for 1 min each; and finally mounted with Permount (Sigma). The immunopositivity of the sections was analyzed with TissueFAXS tissue analysis system (TissueGnostics, Vienna, Austria).

To analyze the immunohistochemical staining numerically, virtual slides were created from the stained samples after scanning each specimen with TissueGenostics system. The TissueFAXS generated true-color digital images of each stained sample, which were analyzed with HistoFAXS software.

### Enzyme-linked immunosorbent assay (ELISA)

*CCL25:* Sera from patients diagnosed with SCC (n=17) or AC (n=14), and from healthy controls (n=9), were provided by Dr. Goetz H. Kloecker of the James Graham Brown Cancer Center, University of Louisville, Louisville, KY. Healthy donors had no active lung disease or symptoms at the time of blood collection. All subjects gave written informed consent. The University of Louisville IRB approved the use of these diagnostic specimens in accordance with the Department of Health and Human Service Policy for the Protection of Human Research Subjects 45 CFR 46.101(b) 2 and use of archived de-identified materials. Serum CCL25 levels were quantified by human CCL25 Quantikine ELISA kit (R&D Systems) according to the manufacturer’s protocol. Briefly, 100 μl of assay diluent (provided with the kit), followed by 50 μl of standards, controls, and serum samples, were added in different wells of an ELISA plate and incubated for 2 h at RT. Following washing four times with Quantikine wash buffer 1 (provided with the kit), 200 μl of conjugate (antibody) was added to each well, and the plate was further incubated for 2 h at RT. The plate was washed, 200 μl of substrate solution was added, and the plate was incubated for 30 min in the dark at RT. Following incubation, 50 μl of stop solution (2N H_2_SO_4_) was added to each well, and the optical density was measured with a microplate ELISA reader at 450 nm with the wavelength correction set at 540 nm. Each sample was tested in duplicate for assessment of inter-assay variation.

*TIMP -1 and -2:* Culture supernatants from CCL25 treated and untreated cells were collected after 24h and levels of TIMP-1 and -2 were quantified by human TIMP-1 and TIMP-2 DuoSet ELISA kits (R&D Systems) following manufacturer’s instructions. Briefly, 100 μl capture antibody (TIMP -1 or -2) were coated in 96 well ELISA plate for overnight at RT. Following washing and blocking, 100 μl of standards, samples and controls were added in duplicates and incubated overnight at 4^0^C. Next day, the wells were washed and 100 μl detection antibody (TIMP -1 or -2) was added in respective wells and incubated for 2 h at RT. After washing, 100 μl streptavidin-HRP conjugate was added and the plate was incubated for 20 min in dark at RT. Following washing, 100 μl of substrate solution was added, and the plate was incubated for 20 min in dark at RT. The reaction was stopped with 50 μl of stop solution (2N H_2_SO_4_), and the optical density was measured with a microplate ELISA reader at 450 nm with the wavelength correction set at 540 nm.

### Cell culture

NSCLC cell lines NCI-H520 (HTB-182, derived from an SCC patient) and NCI-H2126 (CCL-256, derived from an AC patient) were purchased from American Type Culture Collection (ATCC). NCI-H520 cells were cultured in 5% CO_2_ at 37^0^C in RPMI-1640 medium supplemented with 10% fetal bovine serum (HyClone), 100 μg/ml of streptomycin, and 100 U/ml of penicillin (Sigma). NCI-H2126 cells were cultured in 5% CO_2_ at 37^0^C in HITES medium supplemented with 5% fetal bovine serum, 100 μg/ml of streptomycin, and 100 U/ml of penicillin (Sigma). These cell lines were selected as they represent the two NSCLC sub-types, SCC and AC, used in first part of this study.

### Migration and Invasion assays

The migratory and invasive potentials of NSCLC cells were determined with BD Biocoat™ migration and invasion chamber system (BD Biosciences). Briefly, Matrigel inserts were hydrated for 2 h with 500 μl of serum-free DMEM medium at 37^0^C with 5% CO_2_. In the bottom chamber, 100 ng/ml of CCL25 (Peprotech, NJ) was added in serum-free RPMI or HITES medium for SCC (NCI-H520) or AC (NCI-H2126) cells, respectively. The respective culture medium with no CCL25 was used in control wells. Next, 10,000 cells in 500 μl of serum-free RPMI or HITES medium were added per well to the migration and invasion chambers. To determine if the migratory and invasive behaviors of cells were specific to CCL25 gradients, cells were incubated with 1 μg/ml of anti-human CCR9 antibody (R&D systems, MN) for 1 h at 37^0^C with 5% CO_2_ before adding them to the top chamber. The cells were incubated for 16 h and 24 h at 37^0^C with 5% CO_2_ for migration and invasion assays, respectively. Non-migrating cells on the upper surface of the membrane were removed with a cotton swab. The cells that migrated or invaded to the lower surface of the membrane were fixed with 100 % methanol for 3 min at RT, stained with crystal violet for 2 min, and rinsed twice with de-ionized water. The membranes were peeled and mounted on glass slides. The migrated cells were counted by microscopy at 20x magnification. All experiments were repeated three times to validate the results.

### RNA isolation and analysis of mRNA expression

Total RNA was isolated from untreated and CCL25-treated NSCLC cells using Tri-Reagent (Sigma) according to the manufacturer’s protocol. RNA was precipitated and re-suspended in RNA Secure (Ambion, Life technologies, NY). cDNA was generated by reverse transcribing 1 μg of total RNA by use of Verso cDNA Synthesis kits (Thermo Scientific) with random hexamer primers, following the manufacturer’s protocol. Primers specific for CCR9, CCL25, MMP-1, MMP-2, MMP-3, MMP-9, MMP-10, MMP-11, MMP-13, tissue inhibitor of metalloproteinase (TIMP)-1, TIMP-2, and 18S rRNA (internal control) were used to determine mRNA expression with iQ SYBR-Green Supermix (Bio-Rad, CA), as previously described [11]. The real-time thermal cycler (CFX96 Touch, Bio-Rad) profile used for amplification was as follows: initial denaturation 95^0^C for 3 min; denaturation 95^0^C for 30 sec; and annealing, extension, and detection at 60^0^C for 45 sec for 40 cycles. The number of copies for each target was calculated by means of a standard curve, and data were normalized with copies of 18S rRNA in each sample. The results were presented as the number of copies of target gene per 10^6^ copies of 18S rRNA or fold change expression with respect to controls. Gene expression experiments were done in duplicates and repeated three times to validate the results.

### Flow cytometry

Surface expression of CCR9 in NSCLC cell lines was analyzed by flow cytometry. Briefly, cells were washed three times in fluorescence-activated cell-sorting (FACS) buffer (2% FBS in PBS) and treated with 1 μg of Fc Block (BD Biosciences, CA) per 10^5^ cells for 15 min at RT. Fc-blocked cells were incubated with 1 μg of phycoerythrin (PE)-conjugated mouse anti-human CCR9 or PE-conjugated mouse IgG2a isotype control antibodies (R&D System) per 10^5^ cells for 1 h at 4°C. Following staining, the unbound antibodies were removed by washing the cells thrice with FACS buffer. The labeled cells were then fixed in 500 μL of fixative (2% paraformaldehyde in PBS) for 10 min at RT. Fixed cells were washed twice, re-suspended in 500 μl of FACS buffer, and subjected to flow cytometry by FACSARIAII (BD Biosciences, CA, USA). The flow cytometry data were analyzed with Flowjo 10.0.6 software. The experiment was performed in duplicates and repeated three times.

### Gelatin zymography

The activity of MMP-2 and MMP-9 in conditioned medium from LuCa cells treated with CCL25 (0 or 100 ng/ml for 24 h) was analyzed by gelatin sodium dodecyl sulfate-polyacrylamide gel electrophoresis (SDS-PAGE) zymography. Before analysis, cell culture supernatants were concentrated 4-fold by use of Amicon Ultra-4 with a 10-kDa cutoff (Millipore Corporation, MA). Total protein was quantified with BCA protein assay kits (Pierce, Thermo Scientific, IL). Equal amounts of protein (50 μg) were mixed with equal volumes of zymogram sample buffer and resolved on gelatin polyacrylamide gels (Bio-Rad). The gels were incubated in 1x zymogram renaturing buffer (Life Technologies, NY) with gentle agitation for 30 min at RT. Following incubation, gels were equilibrated in 1x zymogram developing buffer (Life Technologies) for 30 min at RT, then incubated at 37^0^C overnight with fresh 1x zymogram developing buffer. Gels were washed three times with deionized water to remove developing buffer and stained with LabSafe GEL blue (G-Biosciences, MO), which revealed gelatinolytic activity as clear bands against a blue background.

To estimate the amount of active MMPs in a sample, the intensity of the lytic bands was analyzed by use of ImageJ software. Data were presented as band areas resulting from the activity of MMP-2 and MMP-9 on the same gel. The experiment was performed using two biological replicates and repeated three times.

### STATISTICS

Comparisons of CCR9 expression immunointensity in lung TMA and comparison of CCL25 levels in serum of healthy controls and NSCLC patients were made by non-parametric Mann Whitney U test. Results were declared significant at α level of 0.05. All *in vitro* experiments were repeated three times. Results of migration and invasion assays and comparisons of MMP-2 and -9 and TIMP-1 and -2 mRNA and/or protein expression were analyzed by non-parametric two-tailed t test; values were declared significantly different at α level of 0.05.

## References

[1] Yang P, Allen MS, Aubry MC, Wampfler JA, Marks RS, Edell ES, Thibodeau S, Adjei AA, Jett J, Deschamps C (2005). Clinical features of 5,628 primary lung cancer patients: experience at Mayo Clinic from 1997 to 2003. Chest.

[2] Montazeri A, Milroy R, Hole D, McEwen J, Gillis CR (2001). Quality of life in lung cancer patients: as an important prognostic factor. Lung Cancer.

[3] Potti A, Abdel-Raheem M, Levitt R, Schell DA, Mehdi SA (2001). Intramedullary spinal cord metastases (ISCM) and non-small cell lung carcinoma (NSCLC): clinical patterns, diagnosis and therapeutic considerations. Lung Cancer.

[4] Spano J-P, Andre F, Morat L, Sabatier L, Besse B, Combadiere C, Deterre P, Martin A, Azorin J, Valeyre D (2004). Chemokine receptor CXCR4 and early-stage non-small cell lung cancer: pattern of expression and correlation with outcome. Ann Oncol.

[5] Svensson M, Agace WW (2006). Role of CCL25/CCR9 in immune homeostasis and disease. Expert Rev Clin Immunol.

[6] Mukaida N, Sasaki S-I, Baba T (2014). Chemokines in cancer development and progression and their potential as targeting molecules for cancer treatment. Mediators Inflamm.

[7] Chen HJ, Edwards R, Tucci S, Bu P, Milsom J, Lee S, Edelmann W, Gümüs ZH, Shen X, Lipkin S (2012). Chemokine 25-induced signaling suppresses colon cancer invasion and metastasis. J Clin Invest.

[8] Singh S, Singh UP, Stiles JK, Grizzle WE, Lillard JW (2004). Expression and functional role of CCR9 in prostate cancer cell migration and invasion. Clin Cancer Res.

[9] Singh R, Stockard CR, Grizzle WE, Lillard JW, Singh S (2011). Expression and histopathological correlation of CCR9 and CCL25 in ovarian cancer. Int J Oncol.

[10] Johnson-Holiday C, Singh R, Johnson E, Singh S, Stockard CR, Grizzle WE, Lillard JW (2011). CCL25 mediates migration, invasion and matrix metalloproteinase expression by breast cancer cells in a CCR9-dependent fashion. Int J Oncol.

[11] Johnson EL, Singh R, Singh S, Johnson-Holiday CM, Grizzle WE, Partridge EE, Lillard JW (2010). CCL25-CCR9 interaction modulates ovarian cancer cell migration, metalloproteinase expression, and invasion. World J Surg Oncol.

[12] Johnson EL, Singh R, Johnson-Holiday CM, Grizzle WE, Partridge EE, Lillard JW, Singh S (2010). CCR9 interactions support ovarian cancer cell survival and resistance to cisplatin-induced apoptosis in a PI3K-dependent and FAK-independent fashion. J Ovarian Res.

[13] Su L, Zhang J, Xu H, Wang Y, Chu Y, Liu R, Xiong S (2005). Differential expression of CXCR4 is associated with the metastatic potential of human non-small cell lung cancer cells. Clin Cancer Res.

[14] Zhu YM, Webster SJ, Flower D, Woll PJ (2004). Interleukin-8/CXCL8 is a growth factor for human lung cancer cells. Br J Cancer.

[15] Phillips RJ, Burdick MD, Lutz M, Belperio JA, Keane MP, Strieter RM (2003). The stromal derived factor-1/CXCL12-CXC chemokine receptor 4 biological axis in non-small cell lung cancer metastases. Am J Respir Crit Care Med.

[16] Saintigny P, Massarelli E, Lin S, Ahn Y-H, Chen Y, Goswami S, Erez B, O’Reilly MS, Liu D, Lee JJ (2013). CXCR2 expression in tumor cells is a poor prognostic factor and promotes invasion and metastasis in lung adenocarcinoma. Cancer Res.

[17] Chen JJW, Yao P-L, Yuan A, Hong T-M, Shun C-T, Kuo M-L, Lee Y-C, Yang P-C (2003). Up-regulation of tumor interleukin-8 expression by infiltrating macrophages: its correlation with tumor angiogenesis and patient survival in non-small cell lung cancer. Clin Cancer Res.

[18] Wang JM, Deng X, Gong W, Su S (1998). Chemokines and their role in tumor growth and metastasis. J Immunol Methods.

[19] Zlotnik A (2004). Chemokines in neoplastic progression. Semin Cancer Biol.

[20] Sivrikoz ON, Doganay L, Sivrikoz UK, Karaarslan S, Sanal SM (2013). Distribution of CXCR4 and γ-catenin expression pattern in breast cancer subtypes and their relationship to axillary nodal involvement. Pol J Pathol.

[21] Wang W-N, Chen Y, Zhang Y-D, Hu T-H (2013). The regulatory mechanism of CCR7 gene expression and its involvement in the metastasis and progression of gastric cancer. Tumour Biol.

[22] Wald O, Shapira OM, Izhar U (2013). CXCR4/CXCL12 axis in non small cell lung cancer (NSCLC) pathologic roles and therapeutic potential. Theranostics.

[23] Rhee Y-H, Chung P-S, Kim S-H, Ahn JC (2014). CXCR4 and PTEN are involved in the anti-metastatic regulation of anethole in DU145 prostate cancer cells. Biochem Biophys Res Commun.

[24] Sekiya R, Kajiyama H, Sakai K, Umezu T, Mizuno M, Shibata K, Yamamoto E, Fujiwara S, Nagasaka T, Kikkawa F (2012). Expression of CXCR4 indicates poor prognosis in patients with clear cell carcinoma of the ovary. Hum Pathol.

[25] Wang L, Wang Z, Liu X, Liu F (2014). High-level C-X-C chemokine receptor type 4 expression correlates with brain-specific metastasis following complete resection of non-small cell lung cancer. Oncol Lett.

[26] Haibi El CP, Sharma PK, Singh R, Johnson PR, Suttles J, Singh S, Lillard JW (2010). PI3Kp110-, Src-, FAK-dependent and DOCK2-independent migration and invasion of CXCL13-stimulated prostate cancer cells. Mol Cancer.

[27] Biswas S, Sengupta S, Roy Chowdhury S, Jana S, Mandal G, Mandal PK, Saha N, Malhotra V, Gupta A, Kuprash DV (2014). CXCL13-CXCR5 co-expression regulates epithelial to mesenchymal transition of breast cancer cells during lymph node metastasis. Breast Cancer Res Treat.

[28] Sharma PK, Singh R, Novakovic KR, Eaton JW, Grizzle WE, Singh S (2010). CCR9 mediates PI3K/AKT-dependent antiapoptotic signals in prostate cancer cells and inhibition of CCR9-CCL25 interaction enhances the cytotoxic effects of etoposide. Int J Cancer.

[29] Ichinose Y, Yano T, Asoh H, Yokoyama H (1995). Prognostic factors obtained by a pathologic examination in completely resected non-small-cell lung cancer. An analysis in each pathologic stage. J Thorac Cardiovasc Surg.

[30] Suzuki K, Nagai K, Yoshida J, Nishimura M, Takahashi K, Yokose T, Nishiwaki Y (1999). Conventional clinicopathologic prognostic factors in surgically resected nonsmall cell lung carcinoma. A comparison of prognostic factors for each pathologic TNM stage based on multivariate analyses. Cancer.

[31] Mirsadraee S, Oswal D, Alizadeh Y, Caulo A, van Beek E (2012). The 7th lung cancer TNM classification and staging system: Review of the changes and implications. World J Radiol.

[32] Saintigny P, Burger JA (2012). Recent advances in non-small cell lung cancer biology and clinical management. Discov Med.

[33] Panse J, Friedrichs K, Marx A, Hildebrandt Y, Luetkens T, Barrels K, Horn C, Stahl T, Cao Y, Milde-Langosch K (2008). Chemokine CXCL13 is overexpressed in the tumour tissue and in the peripheral blood of breast cancer patients. Br J Cancer.

[34] Del Grosso F, Coco S, Scaruffi P, Stigliani S, Valdora F, Benelli R, Salvi S, Boccardo S, Truini M, Croce M (2011). Role of CXCL13-CXCR5 crosstalk between malignant neuroblastoma cells and Schwannian stromal cells in neuroblastic tumors. Mol Cancer Res.

[35] Signoret N, Hewlett L, Wavre S, Pelchen-Matthews A, Oppermann M, Marsh M (2005). Agonist-induced endocytosis of CC chemokine receptor 5 is clathrin dependent. Mol Biol Cell.

[36] Schmalfeldt B, Prechtel D, Härting K, Späthe K, Rutke S, Konik E, Fridman R, Berger U, Schmitt M, Kuhn W (2001). Increased expression of matrix metalloproteinases (MMP)-2, MMP-9, and the urokinase-type plasminogen activator is associated with progression from benign to advanced ovarian cancer. Clin Cancer Res.

[37] Singh S, Singh R, Singh UP, Rai SN, Novakovic KR, Chung LWK, Didier PJ, Grizzle WE, Lillard JW (2009). Clinical and biological significance of CXCR5 expressed by prostate cancer specimens and cell lines. Int J Cancer.

[38] DI Carlo A (2014). Matrix metalloproteinase-2 and -9 and tissue inhibitor of metalloproteinase-1 and -2 in sera and urine of patients with renal carcinoma. Oncol Lett.

[39] Radenkovic S, Konjevic G, Jurisic V, Karadzic K, Nikitovic M, Gopcevic K (2014). Values of MMP-2 and MMP-9 in tumor tissue of basal-like breast cancer patients. Cell Biochem Biophys.

[40] Bourboulia D, Stetler-Stevenson WG (2010). Matrix metalloproteinases (MMPs) and tissue inhibitors of metalloproteinases (TIMPs): Positive and negative regulators in tumor cell adhesion. Semin Cancer Biol.

[41] Schelter F, Grandl M, Seubert B, Schaten S, Hauser S, Gerg M, Boccaccio C, Comoglio P, Krüger A (2011). Tumor cell-derived Timp-1 is necessary for maintaining metastasis-promoting Met-signaling via inhibition of Adam-10. Clin Exp Metastasis.

[42] Fu ZY, Lv JH, Ma CY, Yang DP, Wang T (2011). Tissue inhibitor of metalloproteinase-1 decreased chemosensitivity of MDA-435 breast cancer cells to chemotherapeutic drugs through the PI3K/AKT/NF-кB pathway. Biomed Pharmacother.

[43] Sun J, Stetler-Stevenson WG (2009). Overexpression of tissue inhibitors of metalloproteinase 2 up-regulates NF-kappaB activity in melanoma cells. J Mol Signal.

[44] Gong Y, Scott E, Lu R, Xu Y, Oh WK, Yu Q (2013). TIMP-1 promotes accumulation of cancer associated fibroblasts and cancer progression. PLoS ONE.

[45] Yamashita K, Suzuki M, Iwata H, Koike T, Hamaguchi M, Shinagawa A, Noguchi T, Hayakawa T (1996). Tyrosine phosphorylation is crucial for growth signaling by tissue inhibitors of metalloproteinases (TIMP-1 and TIMP-2). FEBS Lett.

[46] Nemeth JA, Rafe A, Steiner M, Goolsby CL (1996). TIMP-2 growth-stimulatory activity: a concentration- and cell type-specific response in the presence of insulin. Exp Cell Res.

[47] Lizárraga F, Maldonado V, Meléndez-Zajgla J (2004). Tissue inhibitor of metalloproteinases-2 growth-stimulatory activity is mediated by nuclear factor-kappa B in A549 lung epithelial cells. Int J Biochem Cell Biol.

[48] Stetler-Stevenson WG (2008). The tumor microenvironment: regulation by MMP-independent effects of tissue inhibitor of metalloproteinases-2. Cancer Metastasis Rev.

[49] Hoegy SE, Oh HR, Corcoran ML, Stetler-Stevenson WG (2001). Tissue inhibitor of metalloproteinases-2 (TIMP-2) suppresses TKR-growth factor signaling independent of metalloproteinase inhibition. J Biol Chem.

